# Toll-Like Receptor 3 in Liver Diseases

**DOI:** 10.1155/2010/750904

**Published:** 2010-09-23

**Authors:** Shi Yin, Bin Gao

**Affiliations:** ^1^Laboratory of Liver Diseases, National Institute on Alcohol Abuse and Alcoholism, National Institutes of Health, Bethesda, 5625 Fishers Lane, MD 20892, USA; ^2^Department of Geriatrics, The Affiliated Provincial Hospital of Anhui Medical University, Hefei, Anhui 230001, China

## Abstract

Toll-like receptor 3 (TLR3) is a member of the TLR family that can recognize double-stranded RNA (dsRNA), playing an important role in antiviral immunity. Recent studies have shown that TLR3 is also expressed on parenchymal and nonparenchymal cells in the liver as well as on several types of immune cells. In this review, we summarize the role of TLR3 in liver injury, inflammation, regeneration, and liver fibrosis, and discuss the implication of TLR3 in the pathogenesis of human liver diseases including viral hepatitis and autoimmune liver disease.

## 1. Introduction

TLR3 is a member of the Toll-like receptor family that can recognize double-stranded RNA (dsRNA) from viruses, endogenous dsRNA from dying cells, or synthetic dsRNA polyriboinosinic:polyribocytidylic acid (poly I:C). TLR3 expression has been found in endosomal compartments (such as dendritic cells and macrophages) or at the cell surfaces (such as human fibroblasts). Binding of TLR3 and its ligand leads to conformational changes in the TLR3 cytoplasmic tail, followed by recruitment of TIR domain-containing adaptor inducing IFN-*β* (TRIF), and subsequent activation of the mitogen activated protein (MAP) kinase pathway, the NF-*κ*B family of transcription factors, and the IFN regulatory factor (IRF) family of transcription factors, which then induce interferon (IFN) and inflammatory cytokine production [1, 2]. It is generally believed that TLR3 plays an important role in host response to viruses via recognizing dsRNA; however, its role in antiviral immunity has been questioned by some *in vivo* studies [3, 4]. The controversial reports on the role of TLR3 in the antiviral defense may be due to the difference in the type of viruses, the type of cells that are infected, the viral load, its model of infection (endoplasmic versus cytoplasmic), and stage of infection. Recent studies have shown that TLR3 also plays important roles in the pathophysiology of a variety of liver diseases [5–7], which may attribute to the wide expression of TLR3 on all types of liver cells, including hepatocytes [8–10], stellate cells [11], sinusoidal endothelial cells [12], Kupffer cells, biliary epithelial cells [13, 14], as well as immune cells such as NK cells, NKT cells [15], and liver lymphocytes [12]. In this review, we summarize the recent findings regarding the role of TLR3 in liver injury, inflammation, regeneration, fibrosis, viral infection, and autoimmune liver disease. 

## 2. TLR3 in Liver Inflammation and Injury

It has been noticed for many years that injection of mice with the TLR3 ligand poly I:C induced significantly liver inflammation with a predominant accumulation of NK cells [16, 17]. Recent studies suggest that such NK cell accumulation is due to the recruitment of NK cells from the spleen, which is regulated positively by the expression of chemokines and the presence of T cells [18], but regulated negatively by the *β*2 integrin CD11b [19]. Treatment with poly I:C induced mild liver injury in normal mice via an NK cell-dependent manner [20] but induced massive liver necrosis in D-galactosamine (D-GalN)-sensitized mice [21, 22]. In the model of liver injury induced by poly I:C/D-GalN, it is believed that injection of these two reagents elevates the expression of retinoic acid early inducible-1 (Rae-1) on Kupffer cells. Upregulated Rae-1 acts as an NK cell stimulating ligand to activate NK cells via targeting NKG2D receptor on NK cells. Activated NK cells then produce a large amount of IFN-*γ*, which acts in synergy with Kupffer cell-derived TNF-*α* to induce massive hepatocellular damage [22]. In addition, poly I:C treatment also induced significantly liver injury in transgenic mice with HBV surface antigen (HBs-B6) [23, 24]. It was shown that depletion of NK cells or blockage of IFN-*γ* but not depletion of Kupffer cells or neutralization of IL-12 diminished the poly I:C-induced liver injury in HBs-B6 mice, suggesting that NK cells/IFN-*γ* contribute to the pathogenesis of liver injury in this model. In contrast to the detrimental effect of poly I:C on liver injury, pretreatment with poly I:C had a beneficial effect to reduce the mortality and liver injury induced by lipopolysaccharide plus D-GalN in mice [25]. This protective effect of poly I:C seems to be mediated via poly I:C downregulation of TLR4 expression on Kupffer cells/macrophages and subsequent reduction of the responsiveness of Kupffer cells/macrophages to LPS stimulation. In addition, activation of TLR3 on V*α*14 iNKT cells may also negatively regulate liver inflammation via preventing intrahepatic *γ* *δ* T cell accumulation [15].

Although the effect of poly I:C on liver injury has been extensively investigated, the role of TLR3 signaling in these effects remains obscure. TLR3-deficient mice had reduced response to poly I:C stimulation, reduced production of inflammatory cytokines stimulated by poly I:C, and resistance to poly I:C/D-GalN-induced mortality, suggesting a critical role of TLR3 signaling in poly I:C-mediated liver injury [21]. The important role of TLR3 in liver inflammation and injury has also been recently revealed in Concanavalin A- (Con A-) induced T cell hepatitis model by using TLR3-deficient mice [12]. Injection of Con A markedly increased TLR3 expression on liver lymphocytes and sinusoidal endothelial cells. Disruption of the TLR3 gene abolished Con A-induced liver injury. Finally, by using chimeric mice, Xiao et al. [12] demonstrated that TLR3 signaling in both nonhematopoietic and hematopoietic cells plays a critical role in the pathogenesis of Con A-induced T cell hepatitis. However, what the endogenous ligands are and how these ligands activate TLR3 in this Con A-induced T cell hepatitis model remain unknown. 

## 3. TLR3 in Liver Regeneration

Liver regeneration is a very complicated process orchestrated with a series of signaling cascades induced by cytokines, growth factors and hormones [26]. Current knowledge from experimental studies of liver regeneration suggests that tissue loss or cell damage triggers innate immune responses and initiates liver regeneration. Among the multiple innate components, the role of TLR4 signaling in liver regeneration has been extensively investigated. However, the results on the role of TLR4 signaling and its adapter protein myeloid differentiation factor-88 (MyD88) in liver regeneration have been controversial [27, 28]. Early studies showed that TLR4/MyD88 pathway was critical for the initiation of liver regeneration [27]. Impaired liver regeneration was observed in MyD88^−/−^ mice after partial hepatectomy (PHx). This phenomenon was associated with grossly subnormal induction of the expression of immediate early genes involved in hepatocyte replication and the phosphorylation of STAT3 in the liver, and the reduced production of TNF-*α*/IL-6 by the activation of NF-*κ*B in the Kupffer cells. Surprisingly, a later report indicated liver regeneration was not suppressed in mice deficient in MyD88, TLR2, TLR4, or CD14 gene [28]. Although normal hepatocyte DNA replication was observed in Myd88 knockout mice, PHx-mediated induction of proinflammatory cytokines TNF-*α*, IL-6, and their downstream signaling pathways was reduced in MyD-88 knockout mice [28]. At present, the reasons for the discrepancy between these 2 studies are not clear [27, 28]. The different surgical techniques and animal facility environment may contribute to the different findings on liver regeneration in MyD88 knockout mice. 

In contrast, a series of evidence show that TLR3/IFN-*γ*/STAT1 axis plays an inhibitory role in liver regeneration, suggesting that the innate immune system may play an important role in balancing liver regeneration [29–32]. We have previously demonstrated that after infection with murine cytomegalovirus (MCMV) or poly(I:C) injection, NK cells are activated and produce IFN-*γ* that in turn attenuates liver regeneration after PHx. Depletion of NK cells or disruption of either the IFN-*γ* gene or the IFN-*γ* receptor gene enhances liver regeneration and partially abolishes the negative effects of MCMV and poly(I:C) on liver regeneration. These results suggest that viral infection and the TLR3 ligand negatively regulate liver regeneration via activation of innate immunity (NK/IFN-*γ*) [31]. Consistent with these results of the inhibitory role of TLR3 in liver regeneration, TLR3^−/−^ mice demonstrated earlier hepatocyte proliferation and an increase in liver regeneration following PHx [29]. In the absence of TLR3, hepatocyte proliferation was accelerated while the levels of IL-6 and soluble interleukin-6 receptor (sIL-6R) were reduced. TLR3 signaling was induced in hepatocytes at the early time points after PHx, resulting in enhanced NF-*κ*B activation, the increase levels of Rip3 and activation of caspase-8, with no evidence of apoptosis. These findings suggest TLR3 signaling plays an important role in inhibiting liver regeneration [29]. 

We have further demonstrated that IFN-*γ* inhibits liver regeneration via activation of STAT1 and subsequent induction of IRF-1 and p21 [30, 32]. Disruption of the STAT1 gene abolished poly I:C suppression of liver regeneration and the inhibitory effect of poly I:C on liver regeneration was diminished in IRF-1^−/−^ and p21cip1^−/−^ mice. Treatment with IFN-*γ* *in vitro* inhibited cell proliferation of wild-type mouse hepatocytes, but not STAT1^−/−^ hepatocytes. The inhibitory effect of IFN-*γ* on cell proliferation was also diminished in IRF-1^−/−^ and p21cip1^−/−^ hepatocytes, but enhanced in SOCS1^−/−^ hepatocytes. Hepatocyte proliferation was unaffected by treatment with poly I:C alone, but when hepatocytes were cocultured with liver lymphocytes, hepatocyte proliferation was inhibited by IFN-*γ*/STAT1-dependent mechanisms. Moreover, in HCV-infected livers with cirrhosis, activation of STAT1 was detected and correlated positively with liver injury but correlated negatively with hepatocyte proliferation. 

## 4. TLR3 in Liver Fibrosis

Liver fibrosis is a common response to virtually all forms of chronic liver injury and is characterized with hepatic stellate cell (HSC) activation and accumulation of extracellular matrix proteins [33, 34]. HSCs are generally believed to be the most important cells in producing collagens and contributing to the pathogenesis of liver fibrosis. Activation of HSCs is controlled by many cytokines and growth factors. Among them, TGF-*β* is considered the most important factor to induce HSC transformation while PDGF plays a critical role in stimulating HSC proliferation. Recent studies have also suggested that TLR4 plays an important role in promoting liver fibrosis via enhancing TGF-*β* signaling in HSCs [35]. Our laboratory has demonstrated that treatment of mice with the TLR3 ligand poly I:C markedly inhibits liver fibrosis [36, 37], which was confirmed later by another laboratory [38]. The studies from our laboratory as well as other laboratories suggest that multiple mechanisms contribute to poly I:C-mediated inhibition of liver fibrosis. First, poly I:C treatment induces NK cell activation, and activated NK cells then kill early-activated or senescence-activated HSCs that have increased expression of NK cell activating ligands [36, 38]; Second, poly I:C treatment induces NK cells to produce IFN-*γ* that subsequently induces HSC apoptosis and cell cycle arrest [37]. Recent studies from Dr. Schlaak's laboratory have shown that poly I:C treatment stimulates HSCs that express high levels of TLR3 to produce type I IFN-*β* [11]. Since type I IFN is known to inhibit HSC proliferation [39], it is plausible to speculate that activation of TLR3 may also directly block HSC proliferation via production of IFN-*β*, thereby contributing to suppression of liver fibrosis. Furthermore, we have demonstrated that chronic alcohol consumption suppresses poly I:C-mediated activation of NK cell activation and induction of cytotoxic mediators on liver lymphocytes [40] and poly I:C inhibition of liver fibrosis [41]. Alcohol inhibition of poly I:C-mediated activation of NK cells is probably mediated via suppression of poly I:C activation of TLR3 signaling on NK cells [42, 43]. Finally, abrogation of the antifibrotic effect of NK cells by alcohol may be an important mechanism contributing to alcohol acceleration of liver fibrosis in patients with viral infection [41]. We have previously reported that injection of poly I:C inhibits liver regeneration induced by partial hepatectomy [31]. Interestingly, a recent paper shows that injection of poly I:C also inhibits liver regeneration induced by administration of single dose of CCl4 [44]. However, it is not clear whether poly I:C also has inhibitory effect on liver regeneration in a model of liver fibrosis induced by chronic CCl4 treatment, and whether inhibition of liver regeneration by TLR3 also contributes to liver fibrogenesis. 

## 5. TLR3 in Viral Hepatitis Infection

TLR3 is generally believed to play an important role in the innate immune response against viral infection, including viral hepatitis infection, although controversial results have been reported [4]. The antiviral effects of TLR3 signaling on viral hepatitis infection are likely mediated via stimulating of a variety of cells to produce type I IFN that subsequently inhibits HCV or HBV replication [11, 45–47]. These cells include HSCs [11], monocyte-derived dendritic cells [48], hepatocytes [47], Kupffer cells [46], sinusoidal endothelial cells [46], NK cells [49], and so forth. Recent studies showed that TLR3 can also directly sense HCV infection in human hepatocytes, acting independently of retinoic acid-inducible gene I (RIG-I), followed by activation of IRF-3 and ISGs that suppress HCV replication [10]. However, several lines of evidence suggest that TLR3 signaling is suppressed during viral hepatitis infection, which may contribute to the escape of hepatitis virus from the surveillance of innate immunity and lead to the chronic infection. First, expression of TLR3 and type I IFN was significantly decreased in monocyte-derived dendritic cells from patients with chronic HBV or acute-on-chronic HBV liver failure compared with normal healthy individuals. Such reduction correlated positively with severity of the disease [50]; Second, infection of hepatoma cells with HCV *in vitro* degraded TRIF protein, an essential TLR3 adaptor, and subsequently attenuated poly I:C-induced signaling [10]; Third, the NS3/4A serine protease of hepatitis C virus (HCV) can interrupt TLR3 and/or RIG-I-mediated signal transduction by proteolytic cleavage of TRIF and/or CADIF [51–53]. The TLR3 pathway plays an important role in influencing host innate immunity and viral clearance during viral hepatitis infection, and may represent a useful therapeutic approach for the treatment of viral hepatitis. However, a greater understanding of the specific cellular source of TLR3 signals and TLR3 pathway changes in different stages of viral infection may assist in the design of appropriate therapeutic interventions that target this TLR3 pathway in patients with chronic viral hepatitis infection. 

The association between TLR3 gene polymorphisms and chronic HCV infection was also recently investigated [54]. Two single nucleotide polymorphisms (SNPs) were identified within the TLR3 gene: rs5743305 (T/A) is located within the promoter region; rs3775291 (C/T) is located within exon 4. Both SNPs were not found to be associated with TLR3 gene expression in peripheral blood mononuclear cells (PBMCs); however, a tendency of higher TLR3 gene expression in the liver was found for exon 4 TT genotypes. No association was found between both SNPs and the clinical parameters of disease progression of chronic HCV infection, but the TLR3 exon genotype was found to be related to resistance to HCV subtype Ia. These studies suggest that the TLR3 SNP associated with higher TLR3 expression in the liver might be related to the resistance to HCV subtype Ia infection but has no role in disease progression after a chronic infection is established. 

## 6. TLR3 in Autoimmune Liver Disease

TLR3, together with other endosomal TLRs (TLR7 and TLR9), have been implicated in the pathogenesis of a variety of autoimmune diseases [1], including primary biliary cirrhosis. The critical role of TLR3 in autoimmune hepatitis was clearly demonstrated in a model of hepatitis induced by infection of lymphocytic choriomeningitis virus (LCMV) [55]. This study suggests that poly I:C activation of TLR3 on antigen-presentation cells such as macrophages and dendritic cells produce type I IFN and TNF-*α*, which then trigger the release of CXCL9 by hepatocytes, Kupffer cells, endothelial cells, and so forth. CXCL9 then attracts CXCR3 positive self-reactive CD8+ T cells to kill hepatocytes, resulting in autoimmune hepatitis. However, the role of TLR3 in the pathogenesis of human autoimmune diseases is less clear. Immunohistochemistry analyses showed that the expression of TLR3 was markedly increased in biliary epithelial cells at sites of ductular reaction in primary biliary cirrhosis and autoimmune hepatitis [13, 14]. A strong positive correlation between the mRNA levels of TLR3 and type I IFN in the liver was found in the patients with primary biliary cirrhosis, suggesting TLR3 signaling is involved in the pathogenesis of primary biliary cirrhosis [14]. In animal models, injection of poly I:C induced primary biliary cirrhosis-like cholangitis (such as infiltration of mononuclear cells and elevation of AMA autoantibodies) in a genetically susceptible mouse strain of female C57BL/6 mice [56, 57]. At present, the mechanisms by which poly I:C treatment induces cholangitis remain obscure. It is believed that poly I:C activation of TLR3 signaling in biliary epithelial cells as well as hepatocytes and immune cells in the liver results in production of type I IFN, which subsequently contributes to the pathogenesis of primary biliary cirrhosis [13, 14, 58]. However, a recent study suggests that poly I:C induction of type I IFN in biliary epithelial cells is mediated via targeting RIG-I and melanoma differentiation-associated gene 5 (MDA5), not TLR3 [13]. Further studies are required to clarify the role of TLR3 in primary biliary cirrhosis. 

**Figure 1:**
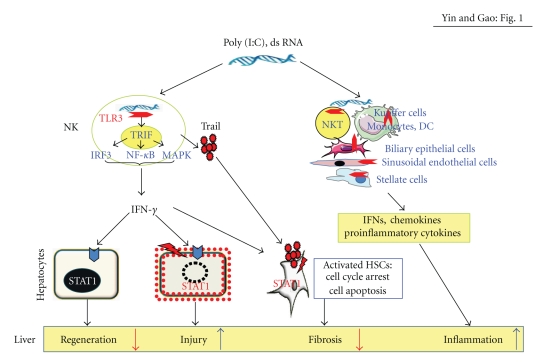
A model depicting the roles of TLR3 in liver injury, inflammation, regeneration, and fibrosis. Poly I:C or dsRNA binds TLR3 receptor in NK cells and stimulates NK cells to produce IFN-*γ* that induces hepatocyte cell cycle arrest and apoptosis via activation of STAT1. IFN-*γ* also induces stellate cell cycle arrest and apoptosis in an STAT1-depdent manner, resulting in inhibition of liver fibrosis. Poly I:C can also inhibit liver fibrosis via activation of NK cell killing of early activated stellate cells. In addition, poly I:C or dsRNA targets TLR3 in many other cell types, followed by production of IFNs, chemokines, and anti-inflammatory cytokines, resulting in liver inflammation.

In summary, TLR3 is expressed on parenchymal and nonparenchymal cells in the liver as well as in many types of immune cells including macrophages, dendritic cells, NK cells, NKT cells, and so forth (see Figure 1). In general, activation of TLR3 by dsRNA induces NK cell accumulation and activation in the liver, leading to liver inflammation and injury. TLR3 signaling also negatively regulates liver regeneration via stimulating NK cells to produce IFN-*γ* that subsequently induces hepatocyte death and cell cycle arrest via an STAT1-dependent manner. Activation of NK cells by the TLR3 ligand poly I:C inhibits liver fibrosis via killing of activated stellate cells and producing IFN-*γ* that subsequently induces stellate cell apoptosis and inhibits stellate cell proliferation. Clinical studies suggest that TLR3 may contribute to the resistance to HCV subtype Ia infection but seems to have no role in disease progression after a chronic infection is established. TLR3 has also been implicated in the autoimmune liver disease in animal models, but more studies are required to clarify the role of TLR3 in human autoimmune diseases. 

## Abbreviations

D-GalN: D-galactosamine
HSC: Hepatic stellate cells
IFN: Interferon
MAPK: Mitogen activated protein kinase
MyD-88: Myeloid differentiation factor-88
Poly I:C: Polyriboinosinic:polyribocytidylic acid (poly I:C)
TLR3: Toll-like receptor 3
TRIF: TIR domain-containing adaptor inducing IFN-*β* (TRIF).
